# MicroRNA-29b Enhances Osteoclast Survival by Targeting BCL-2-Modifying Factor after Lipopolysaccharide Stimulation

**DOI:** 10.1155/2019/6018180

**Published:** 2019-04-10

**Authors:** Ok-Joo Sul, Monisha Rajasekaran, Hyun-Jung Park, Jae-Hee Suh, Hye-Seon Choi

**Affiliations:** ^1^Department of Biological Sciences, University of Ulsan, Ulsan 680-749, Republic of Korea; ^2^Department of Pathology, Ulsan University Hospital, Ulsan 682-714, Republic of Korea

## Abstract

Recent findings suggest that microRNAs (miRs) play a critical role in osteoclastogenesis, which regulates bone loss. We hypothesized that inflammation induces miR-29b, which increases the survival rate in osteoclasts (OCs), leading to bone loss. The expression level of miR-29b increased in OC stimulated by lipopolysaccharide (LPS) in an in vitro system which correlated with its increase in tibiae from mice that received LPS injections compared with those that received vehicle treatment. An miR-29b mimic increased OC survival rate without any change in OC differentiation, and furthermore, the inhibition of endogenous miR-29b induced by LPS decreased OC survival rate. Increased OC survival rate after overexpression of miR-29b was associated with antiapoptotic activity, as shown by staining annexin V-positive cells. We found that a target gene of miR-29b is BCL-2-modifying factor (*Bmf*), which acts as a proapoptotic factor, and that miR-29b binds to the 3′-UTR of *Bmf*. Our data demonstrate that LPS-induced miR-29b increases the number of OC by enhancing OC survival through decreased BMF.

## 1. Introduction

Inflammatory diseases are commonly associated with bone loss, and its underlying mechanisms are complex, interconnected, and mediated through the effects of the bone remodeling cycle. Bone is continuously resorbed by osteoclasts (OCs) and formed by osteoblasts to maintain homeostasis. Bone remodeling is tightly modulated by coordinating bone resorption and deposition, and tipping the balance in either direction results in dysregulation [1]. Excessive bone resorption, defective bone formation, or (most commonly) both are associated with inflammatory disease and lead to bone loss. Bacterial infection is commonly found in inflammatory bone loss [2]. Lipopolysaccharide (LPS) is a component of the bacterial cell wall that acts as a critical pathogen in inflammatory bone loss by acting on both OCs and osteoblasts [3, 4]. LPS can stimulate the production of various osteotropic factors that facilitate OC differentiation and OC activity. In addition, LPS stimulates fusion and increased survival rate [5, 6].

OCs are terminally differentiated multinucleated cells. Bone resorption requires functional OCs and increases the life span of OCs for effective bone loss. Modulation of OC apoptosis has been proposed to reduce bone resorption [7]. OC survival is mainly controlled by the cytokines M-CSF and RANKL, which are generated in neighboring osteoblasts, and various other prosurvival factors. Removal of M-CSF from OC cultures induces caspase and the activation of the MST1 kinase, resulting in apoptosis [8]. However, the other prosurvival factors need to be investigated further to elucidate clearly the mechanism of OC survival.

MicroRNAs (miRs) are ubiquitously found in various organisms [9]. They are the noncoding RNA molecules which act as posttranscriptional regulators via base pairing interactions with sequences in target mRNAs, finally downregulating gene expression by targeting the mRNA for degradation or translational repression of a target gene. Each miR binds multiple target mRNAs, allowing miR to control about one-third of protein-coding genes. Our previous studies and those by other researchers have suggested that miRs play both positive and negative roles in OC differentiation. RANKL-induced miR-183 increases osteoclastogenesis by targeting heme oxygenase [10]. miR-223 and miR-21 promote OC differentiation by repressing NFIA [11] and programmed cell death protein 4 [12], respectively. In contrast, miR-503 and miR-34a inhibit OC differentiation, resulting in protection from osteoporosis, by targeting RANK [13] and transforming growth factor-beta-induced factor 2 [14], respectively. Because miRs act as gene regulatory molecules in the physiological as well as pathological processes of bone, they could be therapeutic or diagnostic targets for ameliorating bone loss. We hypothesized that miR-29b, which can be induced by LPS, might cause bone loss by increasing OC survival rate via targeting of the proapoptotic gene *Bmf*. We report that miR-29b alone inhibited OC apoptosis by decreasing BMF, which led to an enhanced number of OCs.

## 2. Materials and Methods

### 2.1. Ethics Statement

The guidelines of the Institutional Animal Care and Use Committee (IACUC) of the Immunomodulation Research Center (IRC) at the University of Ulsan were applied to all mice we have used in our experiments. All animal procedures were approved by the IACUC of IRC with the approval ID of #UOU-2014-012.

### 2.2. Reagents and Antibodies

LPS and a leukocyte acid phosphatase (TRAP) kit were purchased from Sigma Chemical (St. Louis, MO, USA). Recombinant mouse M-CSF and RANKL were from R&D Systems Inc. (Minneapolis, MN, USA). The mmu-miR-29b mimic (miR-29b mimic), mmu-miR-29b inhibitors (anti-miR-29b), control mimic (con mimic), and control inhibitor (con inh) were obtained from Ambion (Austin, TX, USA). The miScript RT kit and miScript SYBR Green PCR kit were purchased from Qiagen (Hilden, Germany). Lipofectamine 3000 and Lipofectamine™ RNAiMAX reagents were obtained from Invitrogen (Carlsbad, CA, USA). The antibodies used were acquired as follows: primary antibodies against BMF (NBP1-76658) and PUMA (NBP1-76639) from Novus Biologicals (Cambridge, UK), BAK (12105) and BCL-2 (3498) from Cell Signaling (Danvers, MA, USA), BIM (559685) from BD Pharmingen (San Diego, CA, USA), HRK (AHP1178) from Bio-Rad (Oxford, UK), and *β*-actin (A5441) from Sigma Chemical. Primary antibodies against TNF-*α*, IL-1*β*, and IL-6 (AF-410-NA, MAB401, and AF-406-NA, respectively; R&D Systems) were used for neutralization. Small interfering RNA (siRNA) against BMF (sc-45931) and scrambled siRNA (scRNA, sc-37007) were obtained from Santa Cruz Biotechnology (Santa Cruz, CA, USA). Annexin V-FITC, annexin binding buffer, and propidium iodide were purchased from BD Biosciences (San Jose, CA, USA). M-MLV reverse transcriptase, SYBR Green Real-Time PCR Master Mixes, psiCHECK2, and a dual-luciferase reporter assay system were obtained from Promega (Madison, WI, USA). QIAzol reagent was purchased from Qiagen (Hilden, Germany).

### 2.3. Animals, Culture of Osteoblasts and OC, and OC Formation

In the pathogen-free animal facility of IRC, 10-week-old C57BL/6J female mice were housed, and 2 groups of animals were treated with vehicle (phosphate-buffered saline (PBS)) (*n* = 8) and LPS (*n* = 8). LPS was injected once a week (5 mg/kg, i.p.) for 3 weeks as described [15]. The mice were euthanized by CO_2_ asphyxiation immediately. Tibiae that were dissected free of adherent soft tissue were stored at -80°C and homogenized in liquid nitrogen. Then, total RNA was extracted using QIAzol reagent. Primary osteoblasts were prepared from calvarias of C57BL/6J newborn mice as described [15]. Bone marrow cells were obtained from 4-5-week-old C57BL/6J mice as described previously [10]. Further steps were performed following the methods of Sul et al. [15].

### 2.4. miR and siRNA Transfection

BMMs pretreated with M-CSF and RANKL (pre-OCs) were transfected with miR-29 mimic or anti-miR-29 and the corresponding control (con mimic or con inh, respectively) using Lipofectamine 3000 reagent following the manufacturer's instructions. The pre-OCs were transfected with 50 nM siRNA against BMF (siBMF) or with scRNA using 2 *μ*l of Lipofectamine™ RNAiMAX that was diluted with 50 *μ*l of serum-free media. Further steps followed the methods of Sul et al. [15].

### 2.5. RNA Isolation and Quantitative Polymerase Chain Reaction (qPCR)

For RNA preparation and miR qPCR, we followed the methods of Sul et al. [15]. The primer sequences used were as follows: gaccaccttggcaatgtctctg-3′ and 5′-tggctgaggaagtcatctgagttg-3′ (TRAP), 5′- agttgccctcttatgaaggagaag-3′ and 5′-ggagtgtcgtcccagcacat-3′ (calcitonin receptor), 5′-gggccaggatgaaagttgta-3′ and 5′-cactgctctcttcagggctt-3′ (cathepsin K), 5′-ttcagttgctatccaggactcgga-3′ and 5′-gcatgtcatgtaggtgagaaatgtgctca-3′ (ATP6v0d2), 5′-tcctccatgaacaaacagttccaa-3′ and 5′-agacgtggtttaggaatgcagctc-3′ (DC-STAMP), 5′-cccttggggagcagccccctg-3′ and 5′-gccgatggaactggtctgcaa-3′ (BMF), 5′- atattaaccggcgctacgac-3′ and 5′-aggcgatcttggtgaagagt-3′ (BAK1), 5′-gcccagcagcacttagagtc-3′ and 5′- ggtgtcgatgctgctcttct-3′ (PUMA), 5′-cgacagtctcaggaggaacc-3′ and 5′-ccttctccataccagacgga-3′ (BIM), 5′-ggcaagatggacaagacagagg-3′ and 5′-ccaccatgagatctagagagctg-3′ (HRK), 5′-agaccctcacactcagatcatctt-3′ and 5′-ttgctacgacgtgggctaca-3′ (TNF-*α*), 5′-ccagagatacaaagaaatgatgg-3′ and 5′-actccagaagaccagaggaaat-3′ (IL-6), 5′-tcgctcagggtcacaagaaa-3′ and 5′-atcagaggcaaggaggaaacac-3′ (IL-1*β*), and 5′-atcagagagttgaccgcagttg-3′ and 5′-aatgaaccgaagcacaccatag-3′ (RPS).

### 2.6. Apoptosis Assay

OC apoptosis was detected by annexin V staining. BMMs were stimulated with M-CSF and RANKL for 40 h and treated with M-CSF and LPS for 48 h to differentiate mature OCs. Then, mature OCs were washed and incubated with LPS for 6 hours. They were then harvested by incubation with 0.02% EDTA for 20 min (followed by calcium/magnesium-free PBS to scrape the cells), incubated with annexin V-FITC in annexin binding buffer for 15 minutes, and immediately analyzed by FACSCalibur.

### 2.7. Western Blot Analysis

After washing with PBS, the cells were treated with lysis buffer (50 mM Tris-HCl, pH 8.0; 150 mM NaCl; 1 mM EDTA; 0.5% Nonidet P-40; 0.01% protease inhibitor mixture) as described [15]. Cell extracts were loaded on SDS-PAGE and transferred onto the nitrocellulose membrane. To prevent from nonspecific binding, the membranes were treated for 1 h with skim milk in Tris-buffered saline containing 0.1% Tween 20 and were incubated overnight at 4°C with antibodies against BMF, BAK, HRK, BIM, PUMA, BCL-2, and *β*-actin. After washing, the membrane was incubated for 1 h with HRP-conjugated secondary antibodies and developed using chemiluminescence substrates.

### 2.8. Construction of the *Bmf* 3′-UTR Reporter

Fragments containing the *Bmf* 3′-UTR that included the predicted miR-29b binding site were amplified by PCR. The forward primer was 5′-ctcgagggctggccgccctggccggatggatc-3′, and the reverse primer was 5′-gcggccgctgccttaaggtcctcctcaggaccac-3′. The PCR fragment was inserted downstream of the luciferase gene between the Xho1 and Not1 (NEB) sites within the psiCHECK2 luciferase vector, obtaining WT-psiCHECK2-*Bmf* 3′-UTR as described [15]. To obtain constructs with mutated miR-29 binding sites, the miR-29b binding sites in the *Bmf* 3′-UTR element were deleted by PCR splicing. PCR amplification was carried out with WT forward primer 5′-ctcgagggctggccgccctggccggatggatc-3′ and Mut reverse primer 5′-tttcaaattacgattctaccttatggcattgcttt-3′ and Mut forward primer 5′-tcgtaatttgaaatgaaactgtgcacaacataa-3′ and WT reverse primer 5′-gcggccgctgccttaaggtcctcctcaggaccac-3′. The mutated *Bmf* 3′-UTR element with deleted miR-29b binding sites was constructed by crossover PCR. The mutated PCR fragment was inserted into the vector and was named as Mut-psiCHECK2-*Bmf* 3′-UTR. All constructs were verified by sequencing.

### 2.9. Luciferase Assays

A 30 nM miR-29b mimic or anti-miR-29b was used to transfect into RAW264.7 cells with the corresponding control and 700 ng of WT-psiCHECK2-*Bmf* 3′-UTR or Mut-psiCHECK2-*Bmf* 3′-UTR using Lipofectamine 3000 reagent. After 48 hours of transfection, cells were harvested and lysed. A dual-luciferase reporter assay system was used to perform the luciferase assay. Renilla luciferase activity was used to normalize firefly luciferase activity in each sample.

### 2.10. Statistical Analysis

Values are shown as the means of more than triplicate experiments ± SD (*n* = 3~5) with at least three times of repetitive experiments. The student *t*-test was used for comparison between 2 groups or one-way ANOVA that was followed by Bonferroni posttests when multiple groups were compared. The statistical significance was considered with a *p* value of less than 0.05.

## 3. Results

### 3.1. LPS Increases miR-29b in Osteoclasts

**Figure 1:**
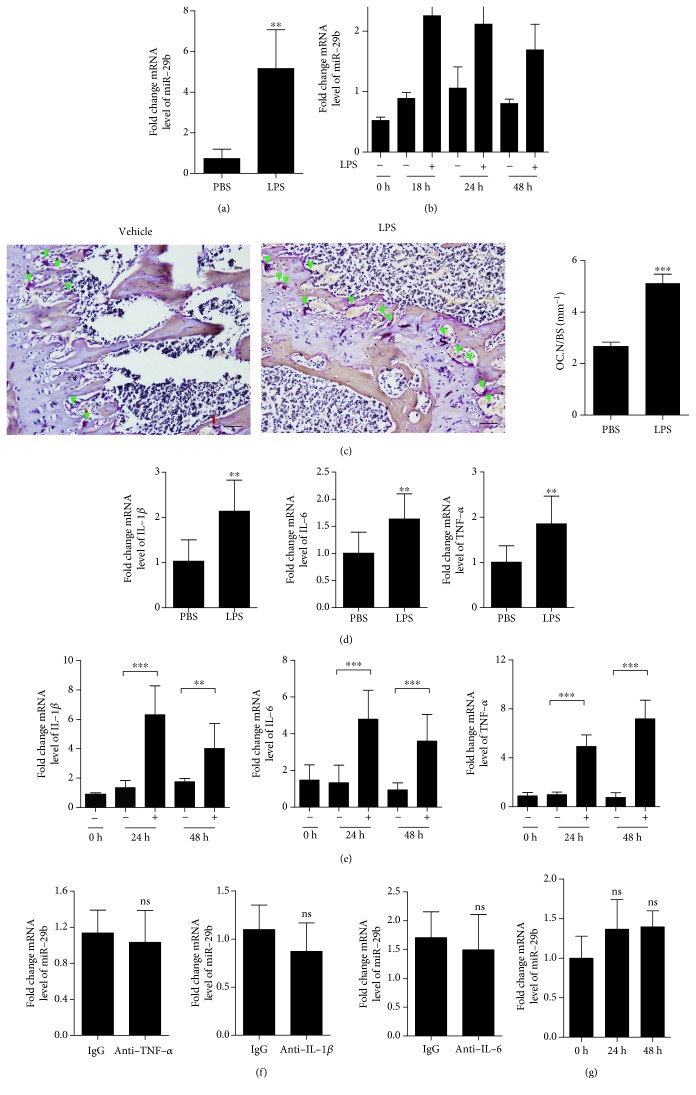
LPS upregulates the expression of miR-29b. Tibiae from LPS-treated or vehicle-treated (V, PBS) mice were analyzed by qPCR to quantify the expression of miR-29b (a) and TNF-*α*, IL-1*β*, and IL-6 (d) (5 mg/kg/d LPS: *n* = 8; V: *n* = 8). BMMs were prepared and incubated with M-CSF (30 ng/ml) and RANKL (40 ng/ml) for 40 h, washed thoroughly, incubated further with LPS (50 ng/ml) in the presence of M-CSF (30 ng/ml) for the indicated times, and then analyzed by qPCR to quantify the expression of miR-29b (b), TNF-*α*, IL-1*β*, and IL-6 (e). (c) To determine TRAP-positive OCs in vivo, mouse femora were excised free of a soft tissue and decalcified in EDTA. Representative histological sections of the distal femoral metaphysis of mice from each of the two groups were stained for TRAP to identify OCs (indicated by the arrowhead) to calculate OC.N/BS. Scale bar: 50 *μ*m in the representative photos. (f) BMMs were prepared and incubated with M-CSF (30 ng/ml) and RANKL (40 ng/ml) for 40 h, washed thoroughly, incubated further with LPS (50 ng/ml) in the presence of M-CSF (30 ng/ml) and each of neutralizing Abs (anti-TNF-*α* Ab, 0.5 *μ*g/ml; anti-IL-1*β* Ab, 2 *μ*g/ml; and anti-IL-6 Ab, 2 *μ*g/ml) or its corresponding IgG (mouse IgG) for the indicated times, and then analyzed by qPCR to quantify the expression of miR-29b. (g) Primary osteoblasts were treated with LPS (50 ng/ml) for the indicated time points and analyzed by qPCR to quantify miR-29b expression. The Ct values of the genes were widely distributed between 17.15 and 28.41 ^∗∗^*p* < 0.01; ^∗∗∗^*p* < 0.001 compared with each corresponding V-treated group. Similar results were obtained in three independent experiments.

To study the role of miR-29b in inflammation-induced bone loss, we determined the expression levels of miR-29b in vivo after LPS administration. In the tibiae of LPS-injected mice, miR-29b expression was about 7.1-fold higher than that in the vehicle-treated mice (Figure 1(a)). Since OCs take part in LPS-induced bone loss [16, 17], we examined whether LPS induces miR-29b in OCs. In the pre-OCs, miR-29b expression was attenuated, but LPS induced an increase of more than 2.5-fold at 18 h, which decreased slightly afterward (Figure 1(b)). As shown in Figure 1(c), OC.N/BS (ratio of OC number to total bone surface area) as evaluated by in vivo TRAP staining was significantly increased in the femur of LPS-treated mice. Since LPS has a tendency to stimulate proinflammatory cytokines including TNF-*α*, IL-1*β*, and IL-6, we wondered whether miR-29b induction after LPS stimulation was mediated by these proinflammatory cytokines. All three cytokines were significantly augmented in the tibiae of LPS-treated mice (Figure 1(d)) as well as OCs stimulated by LPS (Figure 1(e)). Blockade of TNF-*α*, IL-1*β*, and IL-6 using each corresponding neutralizing Ab did not change the expression level of miR-29b (Figure 1(f)), suggesting that miR-29b induction by LPS was not mediated by these proinflammatory cytokines. No significant increase of miR-29b expression was observed in osteoblasts after LPS stimulation (Figure 1(g)).

### 3.2. miR-29b Increases the Number of OCs by Increasing the Survival Rate

**Figure 2:**
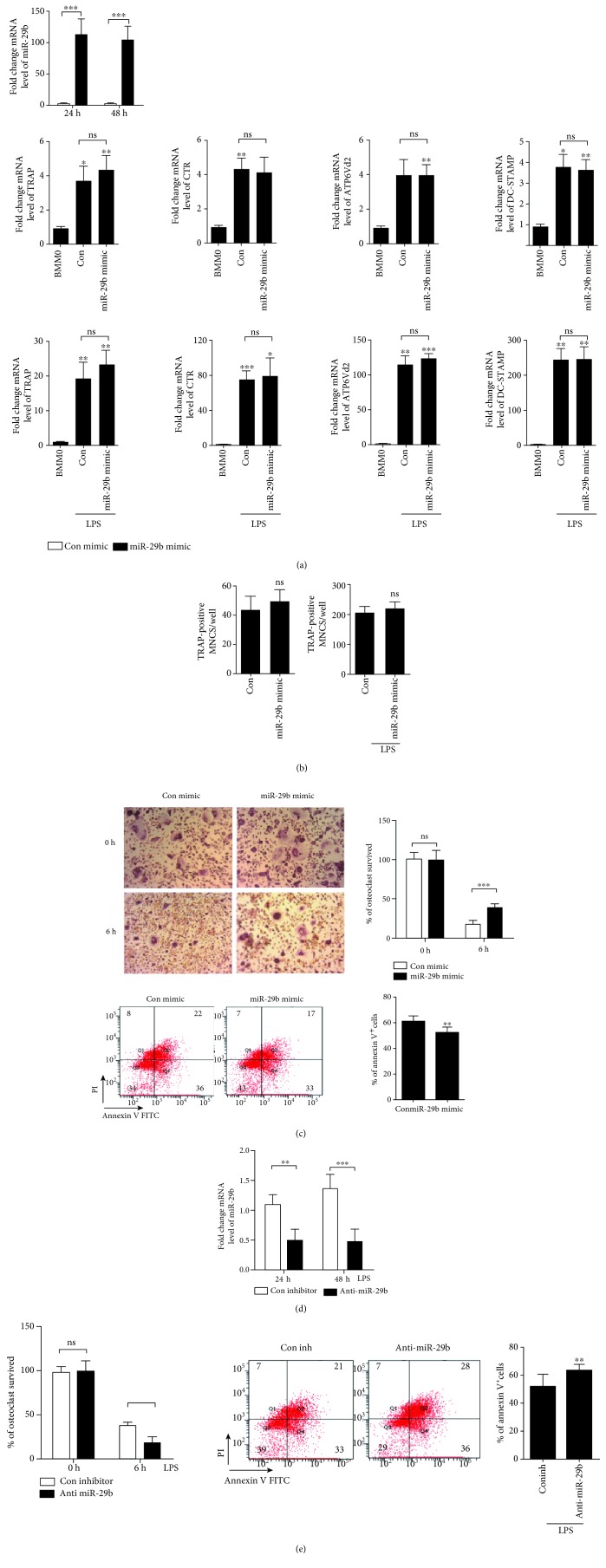
Overexpression of miR-29b increases the number of OCs by increasing the survival rate. BMMs were prepared and incubated with M-CSF (30 ng/ml) and RANKL (40 ng/ml) for 40 h and washed thoroughly. (a, b) Cells were transfected with 30 nM of miR-29b mimic or con mimic in the presence of M-CSF (30 ng/ml) with or without LPS (50 ng/ml). After the indicated times, total RNA was analyzed by qPCR to quantify the expression of miR-29b. Expression levels with con mimic treatment were set at 1 (a). Cells were incubated for 24 h to quantify the expression of TRAP, calcitonin receptor, ATP6v0d2, and DC-STAMP and for 48 h to count TRAP-positive MNCs per well (b). (c) Cells were transfected with miR-29b mimic or con mimic in the presence of M-CSF and LPS. Cells were washed with medium and analyzed after 6 h to measure TRAP-positive MNCs and annexin V-positive cells. (d, e) Cells were transfected with 30 nM of anti-miR-29b or con inh in the presence of LPS (50 ng/ml) and M-CSF (30 ng/ml). After the indicated times, total RNA was analyzed by qPCR to quantify the expression of miR-29b. Expression levels with the con inh treatment were set at 100 (d). Cells were washed with medium, stimulated with LPS for 6 h, and analyzed to measure TRAP-positive MNCs and annexin V-positive cells. Representative photos of OCs. Scale bar: 200 *μ*m. The Ct values of the genes were within 17.82 and 28.65 cycles. ^∗^*p* < 0.05; ^∗∗^*p* < 0.01; ^∗∗∗^*p* < 0.001 compared with each corresponding control. Similar results were obtained in three independent experiments.

To assess the role of miR-29b in osteoclastogenesis, we examined whether the overexpression of miR-29b was enough to induce osteoclastogenesis in the absence of LPS. Compared with the expression following con mimic treatment, transfection of the miR-29b mimic significantly increased the expression level of miR-29b for up to 48 h in pre-OCs (Figure 2(a)). The overexpression of miR-29b, alone or in the presence of LPS, did not change OC differentiation significantly, as demonstrated by the increased number of TRAP-positive MNCs and the expression of OC-specific genes, TRAP, calcitonin receptor, ATP6v0d2, or DC-STAMP, compared to con mimic treatment (Figure 2(b)). Differentiated OCs are subject to apoptosis in the absence of survival factors [18]. LPS increases OC survival when added to mature OCs [6, 19], compared with vehicle, but that effect was lower than that of cytokines. Therefore, we hypothesized that the increased OC survival rate was mediated by LPS-induced miR-29b. To investigate whether miR-29b acts as a survival factor for mature OCs, we examined the effect of the miR-29b mimic on OC survival rate by counting TRAP-positive MNCs after removal of cytokines. As shown in the upper panel of Figure 2(c), the number of OCs surviving after overexpression of miR-29b increased compared to that after treatment with the con mimic. To evaluate whether the increased OC survival rate was caused by an antiapoptotic effect, we examined annexin V staining of mature OCs. The fraction of annexin V-positive cells decreased dramatically after the transfection of the miR-29b mimic compared with that of the con mimic (Figure 2(c)). To show the physiological relevance of miR-29b to OC survival in LPS-stimulated OCs, we used anti-miR-29b to inhibit the expression of endogenous miR-29b in LPS-induced OCs. The transfection of anti-miR-29b in LPS-treated mature OCs significantly attenuated the expression of miR-29b (Figure 2(d)) and decreased the number of OCs when the cytokine was removed, compared with that of con inh (Figure 2(e)). The fraction of annexin V-positive cells was significantly higher after transfection of anti-miR-29b than con inh (Figure 2(e)).

### 3.3. Identification of Target for LPS-Induced miR-29b in OCs

**Figure 3:**
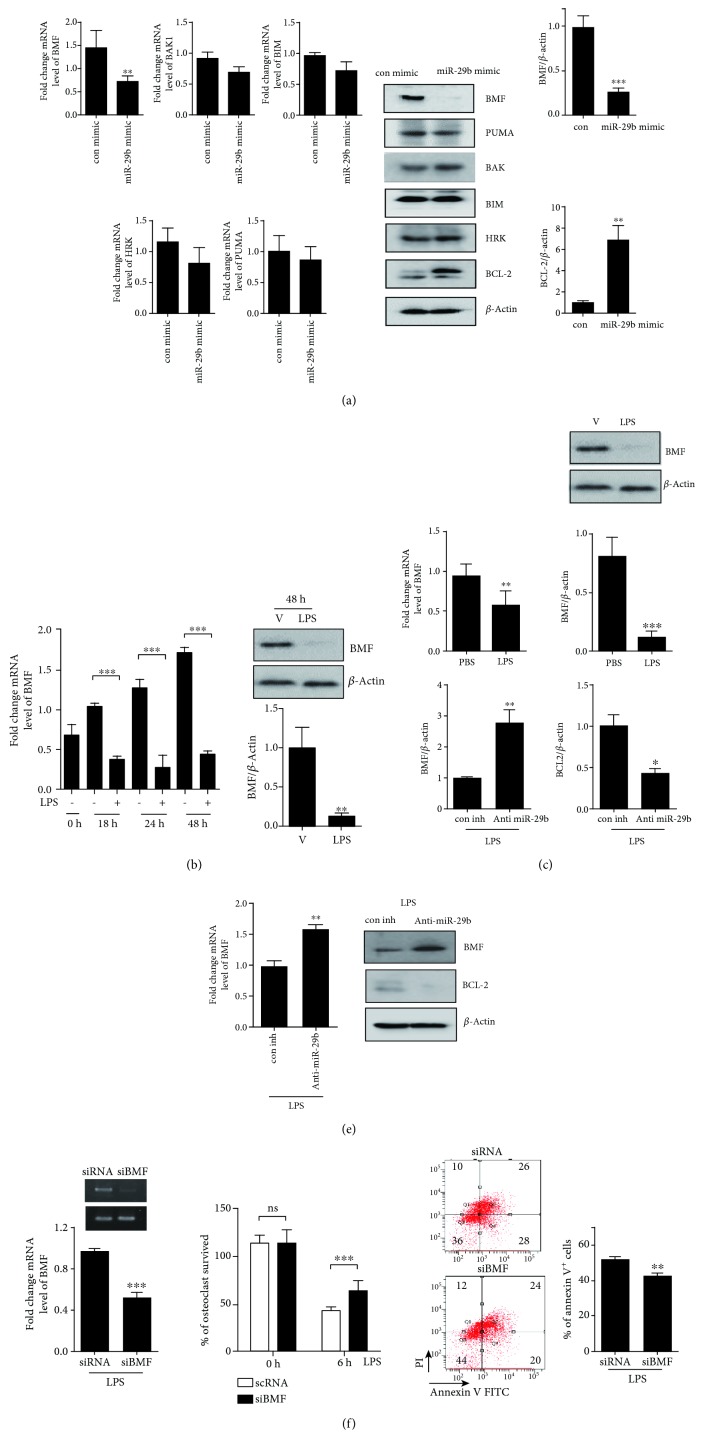
Identification of target for LPS-induced miR-29b in OCs. BMMs were incubated with M-CSF (30 ng/ml) and RANKL (40 ng/ml) for 40 h, washed thoroughly, and incubated further with LPS (50 ng/ml) in the presence of M-CSF (30 ng/ml) for 48 h. (a) Cells were transfected with 30 nM of miR-29b mimic or con mimic in the presence of M-CSF (30 ng/ml) for 6 h. Total RNA was analyzed by qPCR to quantify the expression of BMF, PUMA, BAK1, BIM, and HRK. Expression levels with con mimic treatment were set at 1. Cell lysates were subjected to Western blot analysis with antibodies against BMF, PUMA, BAK1, BIM, HRK, and BCL-2. Antibodies against *β*-actin were used for normalization. (b) Without transfection, total RNA was analyzed by qPCR to quantify the expression of BMF, and cell lysates were subjected to Western blot analysis with anti-BMF Ab. (c) Total RNA and tissue lysate of tibiae from LPS-treated or vehicle-treated (V, PBS) mice were analyzed by qPCR to quantify the expression of BMF and were subjected to Western blot analysis with antibodies against BMF. (d) Cells were thoroughly washed, transfected with 30 nM of anti-miR-29b or con inh, and stimulated with LPS (50 ng/ml) in the presence of M-CSF. After 48 h, total RNA was analyzed by qPCR to quantify BMF expression. Expression levels with con inh treatment were set at 1.0. Cell lysates were subjected to Western blot analysis with anti-BMF and anti-BCL-2 Ab. (e) Cells were thoroughly washed, transfected with 50 nM of scRNA or siBMF, stimulated with LPS (50 ng/ml) in the presence of M-CSF for 48 h, and analyzed to measure TRAP-positive MNCs and annexin V-positive cells. siRNA-mediated silencing of BMF was confirmed by RT-PCR and qPCR. The Ct values of the genes were widely distributed between 17.33 and 30.92. ^∗^*p* < 0.05; ^∗∗^*p* < 0.01; ^∗∗∗^*p* < 0.001 compared with each corresponding control. Similar results were obtained from three independent experiments.

Because the above findings suggest that miR-29b prolonged OC survival by decreasing apoptosis, we next used several websites (http://www.targetscan.org, http://cm.jefferson.edu, http://pictar.mdc-berlin.de, http://mirdb.org/, and http://microrna.org/) to try to identify the molecules associated with apoptosis that might be direct targets of miR-29b. Several regulators of apoptosis, including BCL-2 antagonist/killer 1 (BAK1), BIM, BCL-2 modifying factor (BMF), harakiri (HRK), and p53 upregulated modulator of apoptosis (PUMA), were possible miR-29b targets. To examine the mRNA levels of the potential miR-29b targets, we performed qPCR. Overexpression of miR-29b, but not control miR, resulted in a significant decrease in transcripts of BMF, but not those of PUMA, BAK1, BIM, or HRK (Figure 3(a)). The level of BMF protein also decreased, in agreement with the transcript level, in miR-29b-overexpressing cells, whereas the cellular levels of PUMA, BAK1, BIM, and HRK did not change (Figure 3(a)). Because miR-29b is LPS-inducible, we expected that the miR-29b targets would be attenuated in LPS-induced OCs. The transcript level of BMF decreased significantly after LPS stimulation, and that decrease was sustained for up to 48 h (Figure 3(b)). The expression level of the protein was also reduced 48 h after LPS stimulation (Figure 3(b)). Similar patterns were observed in the tibiae of LPS-treated mice, compared to vehicle-treated mice (Figure 3(c)). Conversely, to reveal the physiological relevance of miR-29b, we assessed whether blocking endogenous miR-29b altered the expression of BMF after LPS stimulation. BMF expression was increased at the mRNA and protein levels, compared to con inh, after transfection of anti-miR-29b in LPS-induced OCs (Figure 3(d)). Next, to examine whether LPS induces OC survival by decreasing BMF, BMF knockdown was performed using siRNA, and OC survival was measured. Downregulation of endogenous BMF increased the percentage of surviving OC and decreased the fraction of annexin V-positive cells (Figure 3(e)).

Because the BH3 domain of BMF is necessary for binding to the prosurvival BCL-2 protein and inducing apoptosis [20], we evaluated the protein level of BCL-2. Overexpression of miR-29b, but not control miR, resulted in a significant increase in BCL-2 proteins (Figure 3(a)), whereas transfection of anti-miR-29b in LPS-induced OCs decreased BCL-2 expression at the protein level compared to transfection of con inh in LPS-stimulated OCs (Figure 3(d)).

### 3.4. miR-29b Directly Interacts with 3′-UTRs of *Bmf*

**Figure 4:**
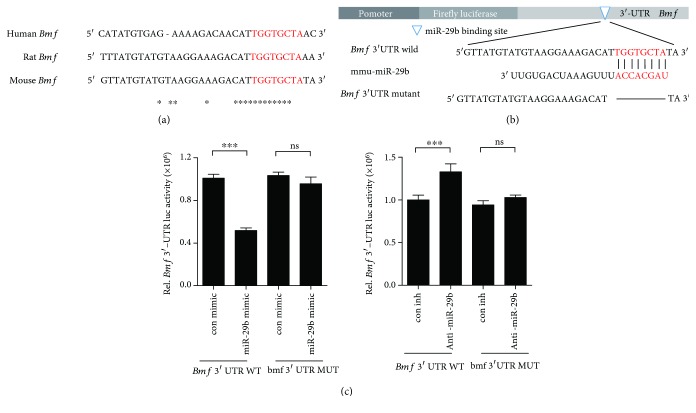
miR-29b directly interacts with *Bmf* 3′-UTRs. (a) The target site of miR-29b in *Bmf* 3′-UTR is conserved in humans and rodents (marked by the star). (b) *Bmf* 3′-UTR mutant was generated by deleting the miR-29 binding site (indicated by the line) to disrupt pairing with mature miR-29b (mmu-miR-29b). (c) Mouse *Bmf* wild-type 3′-UTR containing miR-29b binding sites or its mutated counterpart was cloned into a luciferase reporter vector and transfected into RAW264.7 cells with miR-29b mimic or anti-miR-29b with its corresponding control. Luciferase assays were performed using a dual-luciferase reporter assay system. ^∗∗∗^*p* < 0.001 compared to its corresponding control. Similar results were obtained in three independent experiments.

Finally, we examined whether miR-29b restricts the expression of *Bmf* via direct interaction with 3′-UTRs. In the basis of bioinformatics analysis, the putative miR-29b binding site on *Bmf* 3′-UTR is conserved among species (Figure 4(a)). To assess the interaction between miR-29b and its potential target gene, *Bmf*, mouse *Bmf* wild-type 3′-UTR containing an miR-29b binding site was cloned into a psiCHECK2 vector. A reporter vector containing the mutated *Bmf* 3′-UTR sequence was also cloned (Figure 4(b)). Both of those luciferase reporter vectors and anti-miR-29b were cotransfected into RAW264.7 cells. Anti-miR-29b augmented the luciferase activity compared with con inh, whereas the miR-29b mimic decreased it. However, the luciferase activity was totally regained with the mutated *Bmf* 3′-UTR sequence in accordance with the anticipated target sequence (Figure 4(c)). These results suggest a direct interaction between miR-29b and the 3′-UTR of *Bmf*.

## 4. Discussion

In the present studies, we have demonstrated that LPS increases OC survival rate by inducing miR-29b. Increased expression of miR-29b was found in the tibiae of mice that received LPS injections in vivo as well as in LPS-stimulated OCs in vitro. Overexpression of miR-29b increased the number of surviving OCs by repressing apoptosis, as evaluated by annexin V-positive cells. Conversely, knockdown of endogenous miR-29b induced by LPS attenuated OC survival. Our previous data showed that miR-155 induced by LPS enhanced autophagy and differentiation in OC [15]. miR-155 decreased the expression level of transforming growth factor *β*-activated kinase 1-binding protein 2 (TAB2). Taken together, the effect of LPS on OC appeared to be mediated by the combination of miR-155 and miR-29b that had specific targets to regulate distinct biological functions in OC. Our data showed that LPS did not induce miR-29b in osteoblasts, although the role of miR-29b in osteoblast differentiation has been demonstrated by other findings [21–23] that were in agreement with our previous results that demonstrated that LPS did not change bone formation in vivo [16].

According to the available websites, the potential targets of miR-29b could be proapoptotic molecules such as BAK1, BIM, BMF, HRK, and PUMA. We identified BMF as a target of miR-29b induced by LPS stimulation. Transfection of miR-29b mimic decreased both the mRNA and protein levels of BMF, but it did not affect the levels of the other potential targets. The opposite was observed with transfection of anti-miR-29b in LPS-stimulated OCs. LPS decreased BMF at the mRNA and protein levels in the tibiae of mice which were injected with LPS. And that was the case for the LPS-stimulated OCs. Luciferase assays using plasmids harboring the *Bmf* 3′-UTR showed the interaction of miR-29b and the *Bmf* 3′-UTR. Silencing BMF in LPS-induced OCs resulted in a significant increase in OC survival rate, implying that the BMF action was dominant in LPS-induced OC survival.

miR-29 has been reported as a positive regulator of osteoclastogenesis. In RANKL-induced OCs, the expression of the miR-29 family increases, and knockdown of miR-29 impairs OC commitment and migration without affecting viability, actin ring formation, or survival [24]. The overexpression of miR-29 in THP1 cells dramatically inhibits cell proliferation and induces apoptosis [25]. Enforced expression of miR-29b in K562 cells also inhibits cell growth thereby inducing apoptosis by targeting ABL1 [26]. In contrast, our data show that miR-29b induced by LPS repressed BMF, leading to augmenting OC survival rate through antiapoptotic activity. In agreement with our data, the prosurvival function of miR-29b has been demonstrated by targeting the gene in the proapoptotic BH3 only family in neuronal maturation [27] as well as decreasing BAX expression in doxorubicin-treated cardiomyocytes [28]. These controversial findings suggest that increased miR-29b acts as a positive regulator of OC, but its action depends on its microenvironment.

BMF is a proapoptotic BH3-only protein [29], and the BH3 domain of BMF contributes to both binding to prosurvival BCL-2 proteins to antagonize its activity and inducing apoptosis [20]. We showed that the protein level of BCL-2 was increased by the transfection of an miR-29b mimic, whereas it was decreased by that of anti-miR-29b with LPS. It appears that the expression of the protein that interacted with BMF also changed when miR-29b repressed BMF, although its mechanism of action has not yet been defined.

Taken together, our studies have demonstrated that miR-29b plays a novel role in regulating LPS-induced survival in OCs by targeting BMF. Inhibition of miR-29b expression could be used as a therapeutic target upon inflammation to inhibit OC differentiation by decreasing the life span of OCs.

## Data Availability

The data used to support the findings of this study are included within the article.
